# Artificial Intelligence Models for Zoonotic Pathogens: A Survey

**DOI:** 10.3390/microorganisms10101911

**Published:** 2022-09-27

**Authors:** Nisha Pillai, Mahalingam Ramkumar, Bindu Nanduri

**Affiliations:** 1 Computer Science & Engineering, Mississippi State University, Starkville, MS 39762, USA; 2 College of Veterinary Medicine, Mississippi State University, Starkville, MS 39762, USA

**Keywords:** zoonotic pathogens, mathematical algorithms, machine learning, deep learning

## Abstract

Zoonotic diseases or zoonoses are infections due to the natural transmission of pathogens between species (animals and humans). More than 70% of emerging infectious diseases are attributed to animal origin. Artificial Intelligence (AI) models have been used for studying zoonotic pathogens and the factors that contribute to their spread. The aim of this literature survey is to synthesize and analyze machine learning, and deep learning approaches applied to study zoonotic diseases to understand predictive models to help researchers identify the risk factors, and develop mitigation strategies. Based on our survey findings, machine learning and deep learning are commonly used for the prediction of both foodborne and zoonotic pathogens as well as the factors associated with the presence of the pathogens.

## 1. Introduction

Zoonotic diseases or zoonoses are infections due to the natural transmission of pathogens between animals and humans. Human-animal interactions could lead to the spread of zoonoses by transmission of pathogenic viruses, bacteria, parasites, and fungi through direct or indirect contact, or include vector-borne, food-borne, and water-borne routes. More than 70% of emerging infectious diseases are attributed to animal origin. Thus, zoonoses are a major public health concern with an estimated 2.7 million annual mortality. In addition to their impact on human health, zoonoses impact livestock production and security causing economics losses. Zoonotic diseases can result in epidemics and pandemics exemplified by the recent global coronavirus disease pandemic 2019 (COVID-19) that impacted almost every aspect of life. The World Health Organization COVID-19 dashboard lists 608.3 million confirmed cases and 6.5 million deaths as of September 2022. Early economic projections in 2020 by the United Nations indicated a reduction in global economic output by 8.5 trillion in two years due to COVID-19. Modeling of the impact of climate change and land usage on altered viral-mammal networks predicts at least 15,000 zoonotic spillovers by 2070. Climate hazards are expected to aggravate 58% of known human infectious diseases. While post-outbreak control methods can help mitigate the impact of zoonoses, proactive strategies to identify and mitigate risk are warranted to prevent and reduce the threat to global health, safety, and economy.

In recent years, Artificial Intelligence (AI) models have been used for studying zoonotic pathogens and the factors that contribute to their spread (Carlson et al., 2021 [1]).

In particular, Logistic Regression (Cox 1958 [2]) and Random Forest (Ho 1995 [3], Breiman 2001 [4]) are widely used for modeling and drawing useful inferences about zoonotic diseases and their transmission (Ntampaka et al., 2021 [5], Kiambi et al., 2020 [6], Acharya et al., 2019 [7]). More recently, the effectiveness of artificial neural networks in modeling zoonotic diseases and their causes have also been demonstrated in a number of studies (Boleratz and Oscar 2022 [8], ZareBidaki et al., 2022 [9], Denholm et al., 2020 [10]).

In this review, we provide a summary of AI-based modeling approaches that have been used for zoonotic diseases and pathogens. Throughout this article, we provide information about machine learning (ML) and AI models that are commonly used for analyzing zoonotic pathogen cases, strategies for model selection, and a short summary of results. The scope of this study excludes studies that utilize human or plant-based samples (Buccioni et al., 2022 [11]), or the effects of vaccination (Seekatz et al., 2013 [12]).

The manuscript is organized as follows: Section 2 introduces some fundamental machine learning concepts that are discussed in this paper. In Section 3, we describe the databases and search strings used to identify studies. In the following sections, we examine studies that use artificial intelligence models to address issues concerning zoonotic diseases. We summarize the investigations related to diseases spread by animal contact in Section 4, and food-borne zoonotic pathogens in Section 5. A brief summary of the merits and demerits of popular algorithms included in this manuscript is provided in Section 6. Conclusions are offered in Section 7.

## 2. Artificial Intelligence Models

While mathematical models are useful for scenarios involving a small number of parameters (Schiraldi and Foschino 2022 [13], Adamczewski et al., 2022 [14], Herron 2022 [15]), models based on Artificial Intelligence are especially useful for predicting a wide range of outcomes of interest based on practically any number of parameters—as long as sufficient observations are available to construct such models. Machine learning approaches can be broadly classified into unsupervised approaches for clustering unlabeled data sets, and supervised algorithms for labeled datasets. More recently, with the reduced cost of computation, it has been more useful to categorize them in to traditional machine learning algorithms, which are useful for numerical and category-based inputs, and computationally intensive deep learning algorithms, that can be applied to a wider range of input types, including images and audio.

A brief description of traditional machine learning algorithms widely used in the literature are as follows.

- **K-Nearest Neighbors (K-NN):** A KNN classifier is a non-parametric classifier that uses proximity to determine whether or not an individual data point belongs to a particular group. The nearest neighbors determine the class label by majority vote.
- **Logistic Regression:** It is a parametric, supervised algorithm that uses a logistic (sigmoid) function to model independent variables, viz.,
  $$Y=\frac{1}{1+e^{-W_{X}}}$$
  where *Y* is the dependant variable, $W_{X}$ is the linear combination of independent variables *X* and weights *W*.
- **Random Forest (RT):** A random forest is an ensemble learning technique that constructs an output class through a majority voting approach from a multitude of decision trees.
- **Naive Bayes (NB):** A Naive Bayes classifier is a probabilistic classifier that makes predictions applying Bayes’ theorem, assuming that features are independent.
- **Support Vector Machine (SVM):** Support vector machines are supervised classification algorithms that produce a hyperplane (decision boundary) that separates inputs into different categories.
- **eXtreme Gradient Boosting (XGBoost):** It is an ensemble-based boosting approach that consists of multiple decision trees that run sequentially and are aimed at minimizing the error from the previous model.

The following is a brief description of deep learning models:

- **Artificial Neural Network:** Neural networks are composed of layers of artificial neurons that are processed in a forward direction. This method is intended to identify underlying relationships in a set of data. The system comprises three or more layers: the input layer that accepts the input, any number of hidden layers of neurons, and the output layer that produces the output.
- **Recurrent neural network (RNN):** RNNs are a type of artificial neural network used to address ordinal or temporal problems. Their distinct characteristic is their ability to draw on information from previous inputs to influence current inputs and outputs.
- **Long Short Term Memory network (LSTM):** LSTMs are a special class of RNN with the ability to learn long-term relationships.
- **Generative Adversarial Network (GAN):** A GAN is a supervised deep learning method that learns from the regularities in data. The model is composed of two submodels: a generator model and a discriminator model. A generator model attempts to generate new samples from negative data, while a discriminator model attempts to predict whether a sample is positive or negative.
- **Auto-Encoder:** An autoencoder is an unsupervised method using stacked layers of neural networks composed of an encoder layer, a latent layer, and a decoder layer. By embedding unlabeled data into a latent layer, the original input can be recreated by the decoder layer. A supervised prediction layer can be added to the latent layer to make predictions based on the low-dimensional meaningful representations derived from the input samples.

## 3. Literature Review

An extensive literature review was conducted in accordance with PRISMA guidelines to identify publications related to predictive modeling for zoonotic diseases published between 2015 and 2022. For this study, PubMed, Google Scholar, ACM, IEEE Xplore, ScienceDirect, and BMC were searched for related articles. The following search strings identify studies relating to zoonotic pathogens mentioned in the UNEP and ILRI report 2020 [16] and the Dewey-Mattia et al., 2018 [17].

**String 1:**<Zoonotic_Pathogen> AND *Predictive* AND *modeling*

**String 2:**<Zoonotic_Pathogen> AND <Food_Source> AND *Predictive*

**String 3:**<Zoonotic_Pathogen> AND <Artificial_Intelligence_Model>

**Figure 1:**
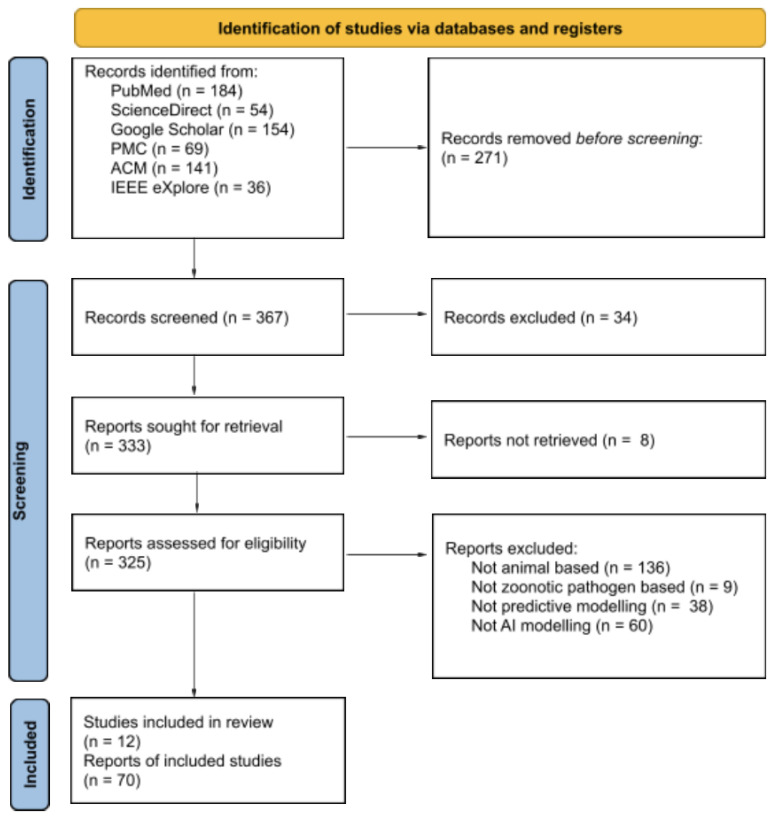
A flowchart illustrating a selection of manuscripts for inclusion in this review based on Preferred Reporting Items for Systematic Reviews and Meta-Analyses (PRISMA).

In the above search strings, <Zoonotic_Pathogen> refers to the bacterium, virus, and parasite names listed in the UNEP and ILRI report 2020 [16] and the Dewey-Mattia et al. 2018 [17]. The term <Food_Source> refers to various animal-based foods, such as milk, chicken, beef, cheese, etc. The term <Artificial_Intelligence_Model>, refers to the widely used machine learning and deep learning models in classification (for example, random forest). Of the 638 publications, 271 were excluded on the basis of their title, 34 papers were excluded based on their abstracts, and 243 papers were excluded after reading the method. Exclusions were made for studies that used human or water samples. In particular, we excluded all studies that were not animal or zoonotic based. Lastly, eligible studies focusing on predictive modeling analysis of zoonotic diseases were included in this review (Figure 1).

## 4. Contact-Based Zoonoses

Studies to investigate zoonotic diseases can be broadly categorized into disease prediction (Section 4.1) and identification of risk factors for prevalence (Section 4.2).

### 4.1. Disease Prediction

Models for predicting incidence of diseases are broadly categorized in to traditional machine learning models (which require only modest computing abilities) and deep learning models.

#### 4.1.1. Machine Learning Models

Using a hybrid **support vector machine** (Cortes and Vapnik 1995 [18]) **and partial least square regression** model, Chinnathambi et al., 2020 [19] effectively forecast trap counts of *Culex Tarsalis*, female mosquitoes that transmit *West Nile Virus*, based on meteorological data, dead birds, WNV cases, and human deaths. Their results show that the **SVM** model, which is based on decision boundaries, works better when classes are separable, outperforms the other machine learning model with a mean absolute error of 3.01.

The **linear regression** model is generally more effective when there is a linear relationship between the variables and the prediction target. Kirjušina et al., 2016 [20] evaluated the larval biomass of naturally infected pine martens (Martes martes) of Latvia using linear regression to investigate the transmission patterns of *Trichinella* spp. from animals to humans. *Trichinella* parasites are cosmopolitan nematodes that infect mainly wild animals. From pine martens that had been infected with *T. britovi*, muscle tissue was collected from the abdomen, back, diaphragm, intercostal muscles, muscles of the head, shoulders, lower and upper parts of the forelimbs and hind limbs, neck, rump and tail, and base and tip of the tongue. Evaluation of larval biomass in reservoir hosts is helpful to predict transmission from carcasses of infected hosts of Trichinella spp. This study estimated the biomass of *Trichinella* larvae from the number of larvae per gram of muscle. According to their results, larvae found in each muscle were able to accurately predict the total larval burden in the animal.

The use of **logistic regression** (Cox 1958 [2]) is demonstrated in Mencía-Ares et al., 2021 [21] as an effective method for determining antimicrobial resistance (AMR) associated with swine farms. The antimicrobial resistance of *Campylobacter*, *Salmonella*, and *Staphylococcus*, the three common zoonotic pathogens in big populations, was assessed for antimicrobial use on swine farm management variables. Univariate mixed-effects logistic regression was used as the machine learning method to assess the influence of production system type, sample type, and antimicrobial consumption on the occurrence of multidrug resistant (MDR) phenotypes. Feces and slurry were sampled for *Campylobacter*; oral fluid was sampled for *Staphylococcus*; and feces, slurry, and oral fluid were sampled for *Salmonella*. This study demonstrated the link between antimicrobial consumption and resistance and concluded that AMR development in *Campylobacter* spp. and *Staphylococcus* spp. is influenced by the production system, with antimicrobial usage as a major factor.

Qekwana et al., 2017 [22] studied patterns and predictors of AMR among *Staphylococcus* spp. isolates from canine clinical samples submitted to the University of Pretoria bacteriology laboratory for routine diagnostic evaluation between 2007 and 2012. The dataset contained 334 confirmed *Staphylococcus* isolates, composed of *S. aureus* and *S. pseudointermedius*, with variables such as the site of collection, breed, sex, age, and the antimicrobial agent used for testing. They explored predictors of AMR in *S. aureus* (98% isolates) and *S. pseudintermedius* (77%) using **logistic regression** models. Chi-square or Fisher’s Exact tests are used to find associations between categorical variables. An analysis of the trends in the proportion of samples resistant to each antimicrobial agent is performed using the Cochran–Armitage trend tests. A binary logistic regression model is used as an initial model to identify antimicrobial resistance predictors from variables such as age, sex, and breed. In the second step, a multivariate logistic regression is conducted using variables identified with a *p*-value less than 0.2 in the first step. Based on the Wald Chi-Square Test, predictor variables with *p*-values less than 0.05 were considered statistically significant. More than 50% of the *S. aureus* isolates tested in their study were resistant to *ampicillin*, *penicillin*, *lincospectin*, and *clindamycin*; more than half of the isolates of *S. pseudointermedius* were resistant to both *ampicillin* and *penicillin*.

Conner et al., 2018 [23] examined AMR predictors among *Staphylococcus* spp. isolated from canine specimens submitted to the University of Kentucky Veterinary Diagnostic Laboratory (UKVDL) between 1993 and 2009. In this study, 4972 *Staphylococcus* isolates were assessed with variables, including the year, *Staphylococcus* spp., geographic region, dog breed, age, group, sex, and specimen source. Cochran–Armitage trend tests were used to analyze the temporal trends for each antimicrobial. AMR and MDR were investigated using **logistic regression** models. This study found 80 isolates of *Staphylococcus* spp. to be resistant to 50% of the antimicrobials tested, while eight isolates were resistant to 75% of the antimicrobials tested. These studies indicate that logistic regression is an effective method for identifying the factors influencing antimicrobial resistance in samples with varying levels of complexity.

*American trypanosomiasis*, or *Chagas disease*, is a neglected tropical disease caused by the *flagellated protozoan*, *Trypanosoma cruzi*. This disease is transmitted by *Haematophagous Triatomines* of the family *Reduviidae*, subfamily *Triatominae*. To detect differences in the intestinal metabolome of the *triatomine Rhodnius prolixus* and predict whether the insect had been exposed to *T. cruzi*, Eberhard et al., 2021 [24] used **logistic regression, random forest** (Breiman 2001 [4]) **classifiers, and gradient boosting** (Friedman 2001 [25]) **algorithms**. Results show that the ensemble approaches outperformed logistic regression for detecting complex interactions between *triatomine* vectors and parasites.

*Ebola virus disease (EVD)* is a rare and deadly disease affecting humans and non-human primates. Using clinical, virologic, and transcriptomic features that distinguish tolerant from lethal outcomes, Price et al., 2020 [26] studied host responses to the *Ebola virus* infection in mice. Based on their analysis, the **random forest** model was found to be capable of accurately predicting disease outcome.

*Crimean-Congo haemorrhagic fever (CCHF)* is a highly virulent human disease caused by a single-stranded, negative sense RNA virus belonging to the genus *Nairovirus* in the family *Bunyaviridae*. Using a structured **Gaussian approach**, Ak et al., 2020 [27] identified risky geographic regions in Turkey for the CCHF (Ak et al., 2018 [28]). The dataset included information on climate, land use, and animal and human populations at risk to capture spatiotemporal transmission dynamics. According to their analysis, CCHF is primarily driven by geographical dependence and climate effects on ticks. The Gaussian process, which is based on a Gaussian probability distribution, can be effectively used to provide reliable classification in uncertain conditions such as climate or spatiotemporal variables.

#### 4.1.2. Deep Learning Models

The advent of neural networks has enabled researchers to derive inferences and make informed decisions from a variety of complex, noisy, and varied datasets from areas including vision, language, audio, and time-series. In one such study, Sadeghi et al., 2015 [29] employed a **neural network** (McCulloch and Pitts 1943 [30]) method for detecting *Clostridium perfringens* infection in chickens based on the characteristics of the sound they produced. The five most important and effective vocal features from the poultry farm were selected based on Fisher Discriminate Analysis (FDA). This study utilized a neural network pattern recognition (NNPR) method to distinguish between healthy and unhealthy chickens by analyzing sound signals, providing new directions for the detection and control of zoonotic pathogens.

Using a hybrid PCA-ANN model, Chenar and Deng 2021 [31] successfully predicted historical outbreaks of oyster *norovirus* along the northern Gulf of Mexico coast. Remote sensing data from the Moderate Resolution Imaging Spectroradiometer (MODIS) satellite, which are gathered at the center of each oyster harvesting area for 10 years, were used as input to this system. Principal component analysis (PCA) was applied to reduce the size of the MODIS Aqua data. The researchers trained an artificial neural network (ANN) model using the first four years’ data, and successfully predicted the outbreaks for six additional years features.

*Avian influenza virus* (HPAI) is a highly contagious virus that belongs to the family *Orthomyxoviridae* and genus *influenza virus A*. Using poultry farm management variables, the visit records of livestock-related vehicles, and environmental variables, Yoon et al., 2020 [32] presented a deep learning model to assess *avian influenza* risk at the farm level. The **multi-layer perceptron** model they developed has proven effective in assessing risk, thus facilitating risk management activities and supporting control measures.

*Bovine tuberculosis* (bTB) is a progressive and debilitating zoonotic disease caused by *Mycobacterium bovis* infection in tissues primarily associated with respiratory tracts and lymph nodes. Denholm et al., 2020 [10] used an ANN architecture to predict the bTB status of UK dairy cows by using mid-infrared spectral profiles, single intradermal comparative cervical tuberculin (SICCT) skin-test results, culture data, and the presence of lesions. The model enabled them to identify cows that are likely to fail the SICCT skin test, which allows farmers to make early management decisions concerning potential reactor cows.

In another case, Cuan et al., 2022 [33] found an effective deep learning method based on a **bidirectional long short term memory neural network** (Hochreiter and Schmidhuber 1997 [34]) for detecting *Newcastle disease Virus*. They extracted complex vocalizations from a specific pathogen-free chicken (SPF) poultry and used them to develop a predictive model to distinguish sick vocalizations from healthy vocalizations.

*Brucellosis* is an infectious disease caused by *brucellae* bacteria that infects the human body and causes symptoms of fatigue, muscle aches, and joint pains. **Convolution-based LSTM recurrent neural networks** were employed by Shen et al., 2022 [35] for epidemic disease prediction using animal stock, food supply information, population, and GDP data. Based on this model, they devised a decision support system for controlling *Brucella*.

The use of neural network models is widespread; however, they are not suitable when the problem does not demand a complex solution. In Arning et al., 2021 [36], popular neural networks such as the **recurrent neural network** and the **long short-term memory network** have been used along with **ensemble** models to determine the source of transmission of *Campylobacteriosis* from a variety of food sources such as chicken, cattle, sheep, and wild birds. The dataset included the whole genome sequences (WGS) and the core genome MLST (cgMLST) of bacteria sampled from infected individuals, contaminated chickens, cattle, sheep, and wild birds. Allelic profiles from MLST, cgMLST, and WGS were encoded as k-mers using DSK (Rizk et al., 2013 [37]). They used the dataset to determine which machine learning algorithm is the most effective for detecting the source of infection. According to their results, tree-based ensemble methods (**random forest and xgboost**) are more effective at predicting the source of human *Campylobacteriosis* with this sample set than more complex neural network models. This highlights the importance of selecting the appropriate algorithm.

Medical management has seen the benefits of deep learning in the prediction of morbidity. Song et al., 2017 [38] developed a **deep denoising autoencoder** (Liou et al., 2014 [39]) to discover the relationship between gastrointestinal diseases and the contaminants. Data were collected from four counties in China that included meat, aquatic foods, and eggs. This study used a denoising auto-encoder with two phases: an encoder that constructs a hidden representation from a noisy input and a decoder that reconstructs the original input in a clean, “repaired” form. A supervised neural network model is also incorporated to predict the presence of contaminants in food. Their analysis showed that deep learning approaches are effective for building predictive models to detect diseases. Their neural network architectures were found to be effective in finding the source of *Campylobacteriosis*, a foodborne illness caused by *Campylobacter jejuni*.

### 4.2. Risk Factors for Pathogen Prevalence

The use of traditional machine learning methods has been instrumental in investigating the relationship between zoonotic diseases and the factors that affect the incidence and distribution of these diseases. Pang et al., 2017 [40] used **logistic regression** (LR) and **random forest** (RF) to analyze the association between meteorological factors and *Listeria* spp. in a mixed produce and diary farm. This study collected fresh cow feces from a dairy barn, cow feed, cow drinking water, and bird feces, and water from the lagoon. A number of meteorological factors were taken into consideration in the analysis, including temperature, precipitation, and wind speed. In both LR and RF models, wind speed and precipitation were found to play a significant role in the transmission of *Listeria* spp. These experiments demonstrate that both models have good predictive capabilities in analyzing risk factors, such as weather.

There is evidence that models based on **logistic regression** are effective for understanding the role of host species in the maintenance and transmission of multihost zoonotic pathogens. González-Barrio et al., 2015 [41] examined the role of European rabbits in the Iberian region as a reservoir for *Coxiella burnetii* using logistic regression models. Serum, spleen, uterus, mammary glands, as well as vaginal, sex, weight, and presence/absence of ruminants and uterus swabs are among the variables examined. The results show that rabbit density plays a major role in the ecology of *C. burnetii*, and that the higher risk of exposure observed during the summer may be the result of increased indirect interactions with *C. burnetii* shed by coexisting ruminants. A subsequent study by González-Barrio et al. 2015 [42] validated the use of multivariate logistic regression models in finding the potential risk factors of *C. burnetii* based on red deer exposure to environmental, host, and management factors.

Due to its ability to determine the importance of features using model coefficients, **logistic regression** is a popular choice for studies involving the impact of livestock farming practices on zoonotic disease transmission. Using samples collected from 100 household clusters with cattle in close proximity to humans, Lupindu et al., 2015 [43] studied the transmission of fecal microorganisms between cattle, humans, water, and soil inside and outside livestock farms, as well as the transfer from livestock farms to the neighborhood. *Ampicillin-* and *tetracycline-resistant Escherichia coli* isolates were detected using logistic regression analysis from cow feces, human stool, soil, and water samples. Using such modeling provides a framework for improving livestock management practices to reduce fecal pollution and the spread of pathogens from livestock manure to humans and the environment. *E. coli* infections associated with pathogens such as *Campylobacter*, *Salmonella*, and *Listeria* were studied by Xu et al., 2022 [44] in pastured poultry farms. For fecal, soil, ceca, and whole carcass rinse processing and chilling samples, a logistic regression model was developed. In their analysis, the amount of *E. coli* in the soil was significantly associated with the predicted presence of *Salmonella*, and the percentage of *Campylobacter* in feces and ceca decreased as *E. coli* concentration increased.

Yoo et al., 2022 [45] used a **Bayesian logistic regression** and an **extreme gradient boosting** model to predict the risk of *Avian influenza virus* occurrence at poultry farms using 12 spatial variables. According to their study, domestic duck farms and the minimum distance to live bird markets were the leading risk factors for outbreaks.

A classification tree may also be used to improve an understanding of interconnected and high-risk groups and their likelihood of contracting disease. Romero et al., 2020 [46] evaluated potential herd-level predictors of *bovine tuberculosis* using **decision trees** and **multivariable logistic regression** in high, edge, and low-risk areas in England. This dataset contained information regarding demographic characteristics of the herd, the history of bTB, cattle movements, badger density, and land class. Using their models, they were able to analyze how bTB risk factors were interrelated to determine the likelihood of an incident occurring in high-risk groups of herds. In addition, Romero et al., 2021 [47] conducted studies using **random forest and LASSO regression** models on the same dataset to identify high-risk farms and develop a targeted disease control strategy.

Even though our survey revealed relatively little use of **Bayesian analyses**, our research has found that Britten et al., 2021 [48] explicitly quantified the advantages of **Bayesian hierarchical modeling** when assisting researchers in selecting the most appropriate methodology to use when collecting heterogeneous environmental data sets. Using **Bayesian models with Laplace approximations** and stochastic partial differential equation, Tumusiime et al., 2022 [49] estimated the risk of *Rift Valley fever* based on animal level factors and meteorological factors. *Rift Valley fever* is a severe viral hemorrhagic fever caused by *RVF virus* (genus *Phlebovirus*, order *Bunyavirales*). Their analyses were based on posterior distributions of model parameters, which enabled them to identify spatial autocorrelation in the data. Their findings concluded that low precipitation, seasonality, haplic planosols, and low cattle density were highly associated with the risk of mortality.

A **random forest**-based predictive model was developed by Hwang et al., 2020 [50] to quantify the relationship between meteorological factors and the presence of *Salmonella* on pastured poultry farms. According to their analysis, the soil model identified humidity as the most significant meteorological variable associated with *Salmonella* prevalence, while the feces model identified high wind gust speed and average temperature as the most significant. In a similar way, Xu et al., 2021 [51] developed a **random forest** predictive model that used farm practices and processing variables to identify variables that can reduce the prevalence of *Campylobacter* on pastured poultry farms.

In recent years, ensemble models have shown success in predicting pathogen presence and evaluating pathogen risk based on a variety of data sets, such as genetic data and remote sensing environmental data. Combining different models to reach an agreeable decision makes ensemble approaches effective when developing predictive models based on nonlinear, imbalanced data. Tsetse flies (family *Glossinidae* and genus *Glossina*), which are obligate parasites and biological vectors of *trypanosomes*, cause human sleeping sickness and animal *trypanosomiasis*. Bishop et al., 2021 [52] used a **random forest regression** algorithm to construct a model for learning about *Glossina pallidipes* habitat suitability across Kenya and northern Tanzania based on genetic data and remotely sensed environmental data. Based on the research, they concluded that vector control will be most successful in the Lake Victoria Basin, and *G. pallidipes* should be managed as a single unit in most of eastern Kenya.

Yoo et al., 2021 [53] employed **Random Forest, Gradient Boosting Machine (GBM), and eXtreme Gradient Boosting** models to predict *avian influenza* using environmental, on-farm biosecurity, meteorological, vehicle movement, and wild bird surveillance data. Eight to ten of the 19 premises infected were predicted to be at high risk in advance by these models. Schreuder et al., 2022 [54] predicted spatial patterns associated with HPAI outbreak risk on Dutch poultry farms based on wild bird density and land cover data. **Random forest** prediction evaluation identified 20 best explaining predictors, of which 17 are water-associated bird species, 2 are birds of prey, and 1 is agricultural cover.

An ensemble approach identified influential factors for prevalence of *Bacillus anthracis*, a soil-borne spore-producing neglected bacterium, is responsible for *anthrax*, an archetypal animal disease. With the use of **artificial neural networks, flexible discriminant analysis, general linear models, general boosted models, classification tree analysis, multiple adaptive regression splines, random forests, and maximum entropy** approaches, Assefa et al., 2020 [55] developed a prediction analysis for *anthrax* using bioclimatic variables, soil characteristic variables, and livestock density variables. Based on their evaluation, the model was influenced by a variety of precipitation factors and animal density factors.

*Creutzfeldt–Jakob disease (CJD)*, also called *mad cow disease*, is a fatal neurodegenerative disease resulting in lesions, cell damage, gliosis, and neuron loss. A popular variant of CJD is caused by consumption of cattle products contaminated with *bovine spongiform encephalopathy (BSE)*. With the use of **elastic net regression, recurrent neural networks, and random forests**, Bhakta and Byrne 2021 [56] learned the predictive causes of the CJD epidemic in the United States. Their results indicated that beer consumption, obesity, and tobacco use are strongly associated with CJD.

Boosting-based ensemble approaches combine weak learners sequentially to improve observations collectively. As a well-known feature selection approach, it is widely used to find features that have a significant impact on the prediction process. It enables the identification of relevant factors involved in the presence of zoonotic pathogens. Prediction of *Aedes mosquitoes* (*A. aegypti* and *A. albopictus*), which belong to the *Flaviviridae* virus family and are the primary vector of the *Zika virus*, utilized **boosted ensemble** approach. Using an ecological network that links *flaviviruses* and their mosquito vectors, Evans et al., 2017 [57] developed a predictive model using **gradient boosted regression** tree to identify associations between vector species and the *Zika virus*. According to their model, 35 species, including **Culex quinquefasciatus and Cx. pipiens**, could transmit the disease. Based on **gradient boosted tree** analysis of wild bird samples, Walsh et al., 2019 [58] predict *avian influenza* viruses. Analysis of sample features, including bird age, sex, bird type, geographic location, and rRT-PCR results, revealed that geographic location and rRT-PCR results are predictive factors.

*COVID-19* is caused by severe acute respiratory syndrome *coronavirus2 (SARS-CoV-2)*, a *coronavirus*. While the origin of *COVID-19 (SARS-CoV-2)* in humans is unknown, using feature vectors derived from spike protein sequences using a position weight matrix (PWM), Ali et al., 2022 [59] assessed the host specificity of *coronaviruses* in birds, bats, camels, swine, humans, and weasels using **boosted regression** algorithms, Fischhoff et al., 2021 [60] combined ecological traits with biological traits to predict the zoonotic potential of *SARS-CoV-2* in greater than 5000 mammals. Based on their results, 540 species belonging to 13 orders were predicted to have a high zoonotic potential for *Coronavirus*.

Based on sequencing of 511 whole genome sequences and 650 spike protein sequences, Brierley and Fowler 2021 [61] developed a **random forest** model to predict the host animal for *SARS-CoV-2*. According to their analysis, human sequences of *SARS-CoV-2* were predicted to have been acquired from bats (suborder *Yinpterochiroptera*), supporting bats as the probable source of the current pandemic.

Using **machine learning algorithms in combination with explainable artificial intelligence** enhances the ability of humans to understand the reasoning behind the decisions made by the AI. Specifically, it enables researchers to explain factors that contributed to a particular prediction. Recently, there has been growing interest in using explanatory tools to investigate the relative importance of biological and ecological factors in pathogen presence. Ndraha et al., 2021 [62] examined the effect of sea surface temperature, precipitation, wind speed, wind gust, salinity, and acidity (pH) on *Vibrio parahaemolyticus* using machine learning and explanatory tools. An **extreme gradient boosting** machine learning algorithm was used to build a prediction model for *Vibrio parahaemolyticus*. According to the results obtained, **XGBoost** is capable of modeling the pathogen in oysters and seawater, but not in sediments. As part of this study, partial dependence plots (PDPs) were generated by **SHapley Additive exPlanations (SHAP)** (Lundberg and Lee 2017 [63]) methods to determine the relationship between environmental variables and the level of *V. parahaemolyticus*. A SHAP dependency plot demonstrates how a single feature impacts the model’s output. According to the relative importance variable analysis, variations in sea surface temperature influence the concentration of *V. parahaemolyticus* in oysters.

Another study (Mollentze et al., 2021 [64]) determined which animal viruses are capable of infecting humans; molecular sequencing data was used to rank pathogens according to their zoonotic potential employing **ensemble methods and SHAP** plots. Bergner et al., 2021 [65] collected metagenomic sequences of feces and saliva from common vampire bats and evaluated their zoonotic potential using **XGBoost**. An analysis of variation in feature importance was performed using **SHAP, and gradient boosted machines (GBMs)** trained on virus taxonomy were used to rank phylogenetic proximity to human-infecting viruses. Based on their findings, 58 viruses were detected as having a higher zoonotic potential, which includes *rabies virus*, *Hepeviridae*, *Coronaviridae*, *Reoviridae*, *Astroviridae*, and *Picornaviridae*.

*West Nile virus* is an emerging arthropod-borne virus that causes *West Nile fever*, which is commonly transmitted by mosquitoes. An analysis of climate factors and regional data was conducted by Wieland et al., 2021 [66] for predicting the distribution of native mosquito species as vectors of the *West Nile virus*. An **XGboost** machine learning algorithm was used for the evaluation model, and the *SHAP* library was used for the identification of explanatory variables. They concluded that regional characteristics play a larger role in the habitat of native mosquitoes than climatic conditions.

Selection of features that influence antimicrobial resistance based on majority voting from diverse AI algorithms is a reliable method for predicting risk factors. Two traditional machine learning approaches (**Random Forest and XGBoost**) as well as three deep learning approaches (**Multilayer Perceptron, Generative Adversarial Network** (Mirza and Osin- dero 2014 [67]), **and Auto-Encoder** Liou et al., 2014 [39]) were used in combination with **SHAP** by Ayoola et al., 2022 [68] to identify critical farm management practices and environmental variables that contribute to multidrug resistance in poultry pathogens in broiler production systems representing background resistance to *Salmonella*, *Listeria*, and *Campylobacter*. A number of recommendations were made in the paper based on the findings in order to mitigate potential multidrug resistance and the prevalence of *Salmonella and Listeria* in pastured poultry.

A **Poisson point process** is another predictive model that assumes independence between samples to be effective. Using wildlife-livestock interfaces, Walsh et al., 2021 [69] examined the landscape epidemiology of *Japanese encephalitis virus (JEV)* outbreaks in India. JEV is a zoonotic disease spread by mosquitoes, particularly *Culex tritaeniorhynchus*. Using a poisson point process, outbreak risk was modeled, which indicated that habitat suitability of ardeid birds and pig density play prominent roles in outbreaks.

Utilizing a **maximum entropy** machine learning model, Walsh et al., 2017 [70] examined the ecological role of wildlife reservoirs and surface water features in the increasing risk of RVF outbreaks. RVF outbreaks were correlated with wetlands, *Bovidae* species richness, and sheep density in their validation study, demonstrating the effectiveness of the maximum entropy machine learning model in learning risk factors. In another study, **MaxEnt model** is used to determine the spatial distribution of exposure, identify environmental parameters, and identified high exposure risk areas for sheep and goats to *C. burnetii* in central Greece Valiakos et al., 2017 [71]. Based on the results of this study, there is a probability of exposure to *C burnetii* of greater than 70% in low altitude zones, irrigated and cultivated agricultural areas, and pastures.

Walsh et al., 2019 [72] evaluated *anthrax*’s geographical suitability in India using a **maximum entropy (Maxent)** machine learning approach that considered both biotic and abiotic factors. There was a significant impact of water–soil balance, soil chemistry, and historic forest loss on the model, and the elephant-livestock interface played a crucial role in the cycle of *anthrax*.

Using a **long short-term memory** model, Tu et al., 2021 [73] assessed the relationship between meteorological factors and population density of *Culex tritaeniorhynchus*. Their analysis showed that mean air temperature and relative humidity had a positive effect on outbreak risk and intensity, suggesting the potential application of neural networks in identifying the factors that influence zoonotic diseases.

A summary of contact-based zoonoses studies, the artificial intelligence model that was used, its application, etiology, and references can be found in Table 1.

## 5. Food-Borne Pathogens

Based on our search, we have observed mainly two types of food-borne zoonotic disease investigations. Based on the surrounding factors, the first approach attempts to predict the presence of food-borne pathogens, while the second case analyzes the dynamics of microbial populations in food.

Numerous factors contribute to the presence of bacteria in food, such as the initial level of contamination, level of nutrients, temperature, pH, activity of the water, and other microorganisms (https://pmp.errc.ars.usda.gov/ (accessed date: 18 September 2022)). It is, therefore, possible to adjust these factors to both prevent food spoilage and ensure food safety. Our literature search did not find any studies that examined the quality of the nutrient medium, so such studies are not included in this review. The growth of microorganisms in foods goes through different phases: the lag phase in which microorganisms adjust to their surroundings, the log or exponential phase in which the population of microorganisms grows exponentially over time, the stationary phase in which the population stabilizes, and the death phase.

Predictive microbiology studies for foodborne pathogens include the estimation of changes in microbial numbers within a production chain under a variety of processing and environmental conditions (McMeekin et al., 2007 [74]). The objective is to determine the number of microorganisms in food at any given point in time to determine the minimum acceptable quality, to determine if the food is safe for consumption, or what treatment can be applied to inactivate the microorganisms. Since microbiological laboratory testing is a time-consuming process, and is not suitable for making quick decisions in real time, predictive microbiology is beneficial for controlling risk and ensuring food safety.

This section presents predictive models and case studies for pathogen prediction (Section 5.1) and bacterial growth dynamics (Section 5.2).

### 5.1. Pathogen Prediction

The purpose of this section is to present case studies that focus on predicting pathogens from food sources. The results of such studies provide valuable guidance for developing a food safety risk management strategy.

Franssen et al., 2017 [75] utilized quantitative microbiological risk assessment (QMRA) methods in their paper to assess the risk of human *Trichinellosis* associated with the consumption of meat from infected pigs, wild boars, and pigs raised in uncontrolled housing. In order to assess the risk model, *Trichinella* muscle larvae, edible muscle types, heat inactivation by cooking and portion sizes, and sensitivity at carcass control are taken into account. To estimate the number of larvae in an animal’s diaphragm, a **negative binomial distribution** is used with maximum likelihood parameter estimation. The beta binomial distribution is used to model the variability associated with *Trichinella* muscle larvae detection. According to the analysis, testing for *Trichinella* in pigs that are kept under controlled housing does not add any value to the protection of human health.

Given the vast array of artificial intelligence techniques available today, choosing the right option for detecting the presence of bacteria can be quite challenging. To detect bacteria such as *Escherichia Coli* and *Staphylococcus Aureus* in raw meat (beef), Amado et al., 2019 [76] employed a variety of machine learning algorithms (**K-Nearest Neighbors, Support Vector Machine, Random Forest, Naive Bayes Classifier, and Artificial Neural Network**). The dataset inputs were derived from the emitted gases of meat. They demonstrated that the Random Forest predictive model achieved the highest level of accuracy (more than 95%) in this classification task, suggesting that ensemble-based models, which combine multiple diverse models to generate a solution, are more effective than single solutions. By comparing the bagging and boosting ensemble techniques, further insight can be gained into the selection of ensemble-based prediction models for the detection of pathogens in food.

Using the core genome multi-locus sequence typing data of *Listeria monocytogenes* isolates, Tanui et al., 2022 [77] compared four popular machine learning approaches (three ensembles) to attribute the source of human *Listeriosis*. The isolates from dairy, fruits, leafy greens, meat, poultry, seafood, and vegetables were used in the dataset. The authors employed supervised classification algorithms, including the **random forest** algorithm (bagging approach), the **support vector machine** radial kernel algorithm, the **stochastic gradient boosting** algorithm (boosting approach), and the **logistic boost** algorithm (boosting approach) in their study. Their analysis found that 17.5% of human clinical cases were caused by dairy products, 32.5% by fruits, 14.3% by leafy greens, 9.7% by meat, 4.6% by poultry, and 18.8% by vegetables. Furthermore, they demonstrated that genomic data combined with machine learning-based models can greatly enhance the ability to track *L. monocytogenes*. Upon analysis, the authors found that the performance of ensemble models did not differ significantly, indicating that any ensemble method would be sufficient to predict pathogens where the data is not highly complex.

### 5.2. Bacterial Growth Dynamics

In this section, we present the case studies for bacterial growth dynamics in foods, the dataset, and a brief conclusion to assist researchers in designing food safety models.

*Salmonella enteritidis* outbreaks that were reported in eleven U.S. states in October, 2018 listed shell eggs as a possible contributing factor (Centers for Disease Control and Prevention 2018 [78]). Based on **Monte Carlo simulation**, Park et al., 2020 [79] developed a predictive model for *Salmonella* spp. and *S. aureus* growth in fresh eggs under isothermal and non-isothermal conditions. However, it has been estimated that there is no likelihood of infection from ready-to-eat egg products due to *Salmonella* spp. or *S. aureus*. Monte Carlo simulation is ideally suited for estimating stochastic and deterministic problems, although poor parameters and constraints could compromise the model’s performance.

In a case study by Dourou et al., 2021 [80], machine learning techniques combined with features derived from Fourier-transform infrared spectroscopy (FTIR) to demonstrate the feasibility of recording the microbiota on foods under dynamic storage conditions. This study focused on *Salmonella*’s ability to survive and proliferate during extended refrigerated storage. They combined **tree-based ensemble methods with support vector regression (SVR)** to estimate the microbial populations in chicken samples. A combination of *Salmonella*-inoculated and non-inoculated chicken liver samples was used for food quality evaluation. The tree-based ensemble approach is used to extract the critical features that best represent the samples, and support vector regression model with radial kernel function is used to estimate *Salmonella* levels. Overall, the results indicated that *Salmonella* was capable of both surviving and growing at refrigeration temperatures.

In **polynomial regression** models, nth degree polynomial transformations of variables are used to approximate the relationship, making it suitable for a wide range of functions. To model the time to detect *Staphylococcal enterotoxins* produced by *Staphylococcus aureus* in cooked chicken products, Hu et al., 2018 [81] proposed a growth predictive model using linear polynomial regression analysis. Assessing the time required to reach the pathogen detection limit could provide valuable insight into food preservation and the quantitative assessment of risk. In this study, the inoculating concentration of *S. aureus* and the incubation temperature were selected as environmental variables. The high correlation coefficient of the regression equation indicated the validity of their methodology. Their study concluded that temperature is the most significant environmental factor that influences the detection of *S. enterotoxins*.

Bulat et al., 2020 [82] measured the differences between microbial load and bacterial shell life of *A. hydrophila* with respect to storage of sardines at different temperatures, using a one-way analysis of variance (ANOVA) to determine differences in daily measures. Sardines’ gills, skin, meat, and intestines were analyzed using statistical prediction models to estimate their shelf-life and quality. According to their findings, sardines stored in the refrigerator had a longer shelf life than those stored at the temperature used for seafood processing. The sardines stored at the temperature used for seafood processing, however, contained higher microbial loads than those stored in the refrigerator.

Summary of food-borne zoonotic pathogen-based studies, research focus, zoonotic pathogen, data source, and references are given in the Table 2.

## 6. Discussion

Models based on artificial intelligence are especially useful for predicting a wide range of outcomes of interest based on practically any number of parameters, as long as sufficient observations are available to construct such models.

Machine learning algorithms such as logistic regression, support vector machines, gradient boosting algorithms, and random forest models are commonly used to predict pathogens and their associated risks. In our literature review, we found studies using these methods, along with linear regression, Naive Bayes, and K-Nearest Neighbors, to identify popular food attributions to diseases. Several popular food choices, such as chicken, beef, pork, dairy products, and seafood, have been found to pose a potential risk factor for various zoonoses based on prediction models. The following are some of the commonly used models, along with their advantages.

- **Support Vector Machine (SVM):** SVM is capable of understanding both the dynamics of population growth for foodborne diseases as well as the prediction of disease and pathogens. It is a memory-efficient algorithm that performs well when there is a clear margin of separation between the samples. It is also capable of handling high-dimensional data. The SVM, however, is not suited to handling large or highly noisy datasets.
- **Logistic Regression:** Several studies have demonstrated the effectiveness of logistic regression as a method for analyzing the influencing factors of zoonotic diseases and those that affect their incidence and distribution. The logistic regression method is suitable for both binary classification as well as multiclassification. In general, it is effective when the data can be separated linearly and the coefficients of the model can be used to determine the importance of the features in the prediction. However, logistic regression does not provide a great deal of insight into nonlinear or complex relationships.
- **Random Forest (RF):** Most studies that employed RF demonstrated that it outperformed other traditional machine learning models. The method is robust to outliers, non-linear data, and high dimensional data. In addition, it is capable of handling unbalanced data and exhibits low bias and variance.
- **eXtreme Gradient Boosting (XGBoost):** Similar to other ensemble approaches, XGBoost is capable of handling outliers, imbalanced data, high dimensional data, and large datasets. The model is less susceptible to overfitting. Research studies have demonstrated that XGBoost paired with SHAP, an explainable AI framework, is an effective methodology for identifying the factors that contribute to the presence of pathogens.

The use of neural networks (deep learning) has been found to be effective for detecting the presence of animal diseases and pathogens in our survey. Multi-layer neural network and long short term memory models have been found to be effective in modeling zoonotic pathogens.

- **Artificial Neural Network:** The ability to model complex, noisy, high dimensional input enables neural network models to effectively use vocal features to distinguish healthy chickens from unhealthy chickens. The use of sound or images in such studies may provide new avenues for the control of diseases. On the other hand, we have found that neural network models are not as effective as ensemble approaches when no complex algorithm is required to learn the data.
- **Long Short Term Memory network (LSTM):** LSTM can be used to address ordinal or temporal problems. LSTM’s distinct characteristic is its ability to draw on information from previous inputs to influence current inputs and outputs. The results of our survey indicate that LSTM can be effectively used for datasets with temporal properties such as food supply, population, and GDP statistics. In situations where the data necessitates the study of spatial or temporal associations, LSTM or RNN can be selected as the algorithm of choice.

**Figure 2:**
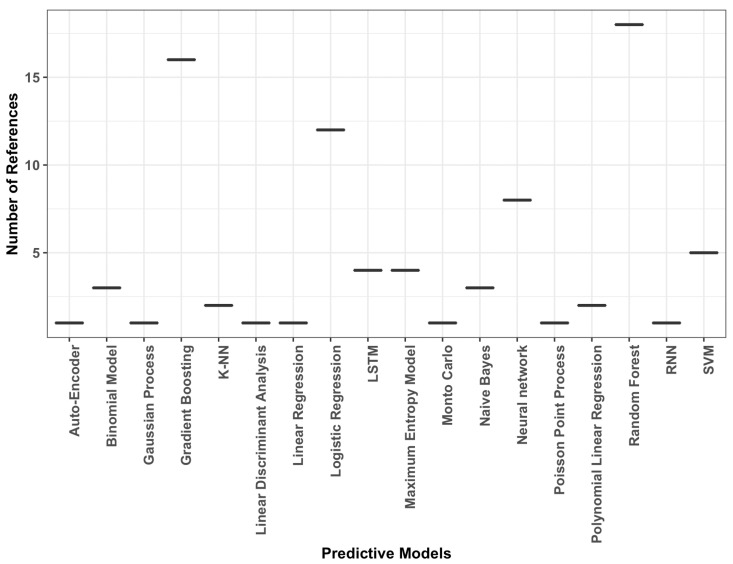
Predictive algorithms and their representation in etiology based studies.

A quantitative representation of predictive algorithms in the literature is presented in Figure 2.

## 7. Conclusions

The aim of this literature survey is to synthesize and analyze machine learning and deep learning approaches applied to study zoonotic diseases. Our review findings will enable researchers to understand predictive models to identify the risk factors for transmission to develop mitigation strategies. The survey revealed that traditional machine learning models are widely used in this field. According to our findings, support vector machines are flexible enough to learn population growth dynamics and predict the occurrence of diseases. With noisy, complex, and varied data, ensemble approaches such as random forest and xgboost have demonstrated excellent performance. However, deep learning methods have tremendous potential for identifying appropriate protective models. Application of deep learning techniques, such as segmentation and classification of images, can enhance research into diagnosing irregularities caused by infections. While the resources in this field are limited, transfer learning (Jeremy et al., 2005 [83]), where we reuse a previously trained model as the basis for training a new model, or zero-shot-based learning (Chang et al. 2008 [84]) that classifies data based on very few or even no labeled examples, have the potential to make learning more efficient and contribute to the development of diagnostic and preventive strategies to limit the spread of zoonotic diseases.

## Figures and Tables

**Table 1:** Summary of AI models and their applications in zoonoses literature.

Model	Application	Etiology	Reference
Logistic Regression	disease prediction	*Campylobacter* *Salmonella* *Staphylococcus*	Mencía-Ares et al., 2021 [21]
	disease prediction	*Staphylococcus* spp.	Qekwana et al., 2017 [22]
	disease prediction	*Staphylococcus* spp.	Conner et al., 2018 [23]
	contamination factor	*Coxiella burnetii*	González-Barrio et al., 2015 [41]
	contamination factor	*Coxiella burnetii*	González-Barrio et al., 2015 [42]
	contamination factor	*Escherichia coli*	Lupindu et al., 2015 [43]
	contamination factor	*Campylobacter* *Salmonella* *Listeria*	Xu et al., 2022 [44]
Random forest	disease prediction	*Ebola virus*	Price et al., 2020 [26]
	contamination factor	*Salmonella*	hwang et al., 2020 [50]
	contamination factor	*Campylobacter*	Xu et al., 2021 [51]
	contamination factor	*Glossina pallidipes*	Bishop et al., 2021 [52]
	contamination factor	*Avian influenza*	Schreuder et al., 2022 [54]
	contamination factor	*SARS-CoV-2*	Brierley and Fowler 2021 [61]
	contamination factor	*SARS-CoV-2*	Fischhoff et al., 2021 [60]
Gradient boosted regression	contamination factor	*Zika Virus*	Evans et al., 2017 [57]
	contamination factor	*Avian influenza viruses*	Walsh et al., 2019 [58]
Poisson Point Process	contamination factor	*Japanese encephalitis virus*	Walsh et al., 2021 [69]
Baysian Model	contamination factor	*Rift Valley fever*	Tumusiime et al., 2022 [49]
Gaussian Process	disease prediction	*Crimean-Congo* *haemorrhagic fever*	Ak et al., 2020 [27] Ak et al., 2018 [28]
Maximum Entropy Model	contamination factor	*Rift Valley fever*	Walsh et al., 2017 [70]
	contamination factor	*C. burnetii*	Valiakos et al., 2017 [71]
	contamination factor	*Anthrax*	Walsh et al., 2019 [72]
Logistic regression, Random forest, Gradient boosting	disease prediction	*Trypanosoma cruzi*	Eberhard et al., 2021 [24]
Logistic regression, Random Forest	contamination factor	*Listeria* spp.	Pang et al., 2017 [40]
Linear Regression	disease prediction	*Trichinella* spp.	Kirjušina et al., 2016 [20]
Support Vector Machine, least square regression	disease prediction	*Culex Tarsalis*	Chinnathambi et al., 2020 [19]
XGBoost SHAP	contamination factor	*Vibrio parahaemolyticus*	Ndraha et al., 2021 [62]
	contamination factor	*Rabies virus*, *Hepeviridae*, *CoronaviridaeReoviridae*, *Astroviridae*, *Picornaviridae*	Bergner et al., 2021 [65]
	contamination factor	*West Nile virus*	Wieland et al., 2021 [66]
Artificial Neural Network	disease prediction	*Clostridium perfringens*	Sadeghi et al., 2015 [29]
	disease prediction	*Norovirus*	Chenar and Deng 2021 [31]
	disease prediction	*Avian influenza virus*	Yoon et al., 2020 [32]
	disease prediction	*Bovine tuberculosis*	Denholm et al., 2020 [10]
Long short term memory	disease prediction	*Newcastle disease Virus*	Cuan et al., 2022 [33]
	disease prediction	*Brucellosis*	Shen et al., 2022 [35]
	contamination factor	*Japanese encephalitis virus*	Tu et al., 2021 [73]
Long short-term memory network, XGboost Recurrent neural network, Random forest	disease prediction	*Campylobacteriosis*	Arning et al., 2021 [36]
Auto-Encoder	disease prediction	*Campylobacteriosis*	Song et al., 2017 [38]
Bayesian logistic regression, XGBoost	contamination factor	*Avian influenza virus*	Yoo et al., 2022 [45]
Decision trees, Logistic regression	contamination factor	*Bovine tuberculosis*	Romero et al., 2020 [46]
Random Forest, LASSO regression	contamination factor	*Bovine tuberculosis*	Romero et al., 2021 [47]
Random Forest, XGBoost	contamination factor	*Avian influenza*	Yoo et al., 2021 [53]
Neural Network, Random forest, Maximum Entropy	contamination factor	*Anthrax*	Assefa et al., 2020 [55]
Recurrent neural network, Random forest	contamination factor	*Creutzfeldt-Jakob disease*	Bhakta and Byrne 2021 [56]
Random Forest, XGBoost, Multilayer Perceptron Generative Adversarial Network, Auto-Encoder, SHAP	contamination factor	*Salmonella*, *Listeria*, and *Campylobacter*	Ayoola et al., 2022 [68]

**Table 2:** Summary of models and their applications in foodborne zoonoses.

Model	Application	Etiology	Datasource	Reference
Monte Carlo simulation	population growth	*Salmonella* spp.	fresh eggs	Park et al., 2020 [79]
Support Vector Regression	population growth	*Salmonella* spp.	chicken	Dourou et al., 2021 [80]
Polynomial Regression	population growth	*Staphylococcus aureus*	chicken	Hu et al., 2018 [81]
K-Nearest Neighbors, Support Vector Machine, Random Forest, Naive Bayes Classifier and Artificial Neural Network	pathogen detection	*Escherichia coli* *Staphylococcus aureus*	beef	Amado et al., 2019 [76]
Random forest, Support vector machine, Radial kernel, Stochastic gradient boosting, Logistic boost	pathogen detection	*Listeria monocytogenes*	dairy, fruits, leafy greens, meat, poultry, seafood	Tanui et al., 2022 [77]

## Data Availability

Not applicable.
